# ‘Evolution-Proofing’ Antibacterials

**DOI:** 10.1093/emph/eou020

**Published:** 2014-08-13

**Authors:** Adin Ross-Gillespie, Rolf Kümmerli

**Affiliations:** Microbial Evolutionary Ecology, Institute of Plant Biology, University of Zürich, Winterthurerstrasse 190, 8057 Zürich, Switzerland

## Antibiotic resistance: inevitable?

When antibiotics first came into use, they were so effective at curbing bacterial infections that it seemed the age-old battle of man *vs.* microbe would soon be at an end [1]. Eighty years and dozens of drugs later, we now know better. Following each new antibiotic’s launch, reports soon accumulated that once-treatable infections were becoming refractory to the drug [2]. Nowadays, many infectious strains are already resistant to multiple antibiotics [3]. Treating such cases is becoming more and more difficult, expensive and risky. Meanwhile, the supply of new antibiotics has stalled. All this adds up to a major global crisis. It is already underway, and it is worsening every day [3].

The rise of resistance is simply adaptation—evolution in action. Bacteria’s large populations and their proclivity for swapping genes mean that mutants arise regularly, and thereafter, the fittest mutants spread through natural selection. So is resistance wholly inevitable? Not necessarily! Evolutionary theory not only explains why resistance occurs but it also offers clues as to how we might be able to prevent it—or at least slow it.

## Evolutionary perspectives

First, we can try to reduce the risk of resistance arising in the first place [4]. To resist a single antibiotic, one mutation might suffice, but to resist a ‘cocktail’ of distinct drug types, more complex suites of mutations may be needed—the odds of which should be lower. We can also narrow the range of potential ‘routes to resistance’. In bacteria, resistance most typically involves changes on or within cells - blocking a drug’s entry, expelling or degrading it before it can act, or altering its intracellular target [5]. Drugs that act outside the cell may thus be less likely to elicit resistance-conferring mutations [6].

However, resistance can still arise, so we should try to minimise its spread [4]. One approach is to use drugs that curb bacterial virulence but not growth. Mutants resistant against such drugs, if they arise, should have no growth advantage over susceptible types. Another approach would be to target the secreted virulence factors that are shared cooperatively among co-infecting bacteria. Mutants able to maintain production of the shared virulence factors would benefit both resistant and susceptible variants alike, and so should have no selective advantage [4, 6].

## Future implications

Do these ideas for ‘evolution-proofing’ have empirical support? Combination therapy has been used for years, yet data suggests that only certain drug mixes work—and only for certain infections [7]. More recent ideas, however, may hold greater promise. Examples include: (i) preventing adhesion to host tissue [8]—a therapy that acts extracellularly; (ii) inhibiting communication among bacteria [9]—a therapy inhibiting the collective release of sharable toxins; and (iii) the extracellular quenching of iron-binding molecules [6]—another therapy that, by targeting a social trait, curbs the growth of resistant and susceptible bacteria alike. ‘Evolution-proof’ therapies may thus already exist, but more work is needed—urgently—if they are to make their way into the clinic.
